# Determining the factors affecting customer satisfaction using an extraction-based feature selection approach

**DOI:** 10.7717/peerj-cs.850

**Published:** 2022-01-25

**Authors:** Weishen Wu, Dalianus Riantama

**Affiliations:** 1 Department of Information Management, Da-Yeh University, Changhua, Taiwan; 2 College of Management, Da-Yeh University, Changhua, Taiwan

**Keywords:** COVID-19, Online travel agencies, Text mining, LASSO, Feature selection, Customer satisfaction

## Abstract

The coronavirus disease 2019 (COVID-19) causes tremendous damages to the world, including threats to human’s health and daily activities. Most industries have been affected by this pandemic, particularly the tourism industry. The online travel agencies (OTAs) have suffered from the global tourism market crisis by air travel lockdown in many countries. How online travel agencies can survive at stake and prepare for the post-COVID-19 future has emerged as an urgent issue. This study aims to examine the critical factors of customers’ satisfaction to OTAs during the COVID-19 pandemic. A text mining method for feature selection, namely LASSO, was used to deal with online customer reviews and to extract factors that shape customers’ satisfaction to OTAs. Results showed that refunds, promptness, easiness and assurance were ranked as the most competitive factors of customers’ satisfaction, followed by bad reviews & cheap and excellent service & comparison. New factors to customers’ satisfaction were revealed during the global tourism recession. Findings provide OTAs guidelines to reset services priorities during the pandemic crisis.

## Introduction

Online travel agencies (OTAs) are online business that facilitates customers to purchase travel, hospitality, and tourism products/services from providers (*e.g.*, airlines, hotels, rental cars, restaurants, cruises, package holidays, etc.) and gets commission charged on transactions as an intermediary (Long & Shi, 2017). During the coronavirus disease 2019 (COVID-19) pandemic worldwide in 2020, OTAs have been hit hard (Sigala, 2020). Given the negative impacts on the tourism industry in such a crisis, customer satisfaction turns out to be crucial thus real-time research on it is desperately demanded (Sharma, Sharma & Chaudhary, 2020; Zhang, Hou & Li, 2020). Several OTAs started to cooperate with tourism suppliers to promote customer satisfaction during the COVID-19 pandemic (Hao, Xiao & Chon, 2020).

Most of the studies adopted questionnaires to obtain customers’ voices to measure the factors that influence customer satisfaction in various tourism contexts, such as OTAs (Hao et al., 2015; Rajaobelina, 2018), hotels (Davras & Caber, 2019; Nunkoo et al., 2020), airlines (Tahanisaz & Shokuhyar, 2020) and restaurants (Gopi & Samat, 2020). For the survey method, respondents may not pay attention to every item or randomly answer questions resulting in incomplete data (Evans & Mathur, 2018). This study uses online customer reviews (OCRs) that can lower the inaccuracy of artificial responses given by customers to questionnaire surveys (Sánchez-Franco, Navarro-García & Rondán-Cataluña, 2019). OCRs are the user-generated content containing text comments and rating scores of companies or brands that are posted on e-vendor websites or third-party websites (Mudambi & Schuff, 2010). Few researchers utilized OCRs in the domain of online travel agencies but not to understand customer satisfaction (Hou et al., 2019). In addition, the questionnaire survey demands researchers to identify the satisfaction’s factors in advance whereas a gap between what researchers and managers believe is important and what customers say is important in the evaluation and selection of services (Lockyer, 2005). In contrast to previous studies, instead of identifying customer satisfaction’s factors in advance, this research goes differently to search for customer satisfaction’s factors blindfold. This study uses OCRs to apply exploratory research to understand customer satisfaction’s factors, and the outcomes can be more reliable. Exploratory research is suitable for this study because we believe that we could not use our past knowledge to judge for specific situations such as COVID-19 circumstances.

Drawing from the literature on impression formation, it is important that researchers further investigate the first question of what causes customer satisfaction among OTAs’ customers. To the OTAs, customer satisfaction is an antecedent to customer loyalty, repurchasing, and positive/negative OCRs (Rianthong, Dumrongsiri & Kohda, 2016; Long & Shi, 2017; Cui, Lin & Qu, 2018; Brun et al., 2020; Sharma, Sharma & Chaudhary, 2020). The second question of what is from the most to the least important ranked of customer satisfaction’s attributes. As a result, OTAs can better set priorities for the attributes that are most important to customers while also improving cost performance. By answering those questions, this study contributes to the literature in two respects. This study aims to reveal and rank the significant factors of OTA customer satisfaction during the COVID-19 outbreak.

The remainder of this article is organized as follows. This study first presents an outline of the foundation of this examination and relevant literature. This study extracts OCRs and adopts a text mining approach to deal with them. Next, this research investigates customer satisfaction’s factors using a multimethod approach applying big data sets from the largest OTA in the world. Finally, this study discusses the key findings and practical implications for OTAs and considers future examination necessities.

## Literature Reviews

### Customer satisfaction toward online travel agencies

The concept of customer satisfaction covers the expectation/disconfirmation paradigm, the norm view, the equity view, and the perceived overall performance (Yoon & Uysal, 2005). The theoretical foundation of this research is based on expectation/disconfirmation theory. There are two scenarios for expectation/disconfirmation theory: affirmation (satisfaction) if the perceived outcome meets expectations; and negative disconfirmation (dissatisfaction) if expectations are not reached (Yüksel & Yüksel, 2001). Previous studies show that factors influencing customers’ pre-purchasing expectations consist of product- and service-related factors and customer-related factors. In the context of OTA, the product- and service-related factors include website reputation, available choices, and product price (Chang, Hsu & Lan, 2019) and influence customer expectations (Ha & Janda, 2016; Kim et al., 2020).

Service quality attributes were the most factors analyzed by previous studies to understand customer satisfaction as shown in Table 1, largely ignoring external factors. It is unknown whether external factors have an impact on customer satisfaction toward OTAs. Nowadays, the hospitality and tourism industry is very influenced by the rapid development of information technology. The internet makes external factors such as online customer reviews hold a big portion to affect customer satisfaction (Sharma, Sharma & Chaudhary, 2020; Wang et al., 2020).

### Online customer reviews

OCRs provide a rich source of data to extract the dimensions of customer satisfaction for tourism sectors (Chen et al., 2019; Hlee et al., 2020; Joung, Kim & Kim, 2021; Lien, Wen & Wu, 2011; Zinko et al., 2021). The results of the studies using OCRs ought to be more dependable and exact than those statistical results acquired from conventional satisfaction surveys dependent on little data samples (Sánchez-Franco, Navarro-García & Rondán-Cataluña, 2019). In addition, when the social distancing was carried out in the pandemic, readers’ perceptions toward certain products or services mainly relied on OCRs (Hernández-Ortega, 2018).

**Table 1:** Review of literature on satisfaction in tourism sectors.

References	Context	Factors/Predictors/Antecedents	Findings
Kim & Lee (2004)	OTA	Structure & ease of use, information content, usefulness & reputation, and security	Information content was found to be the most important factor in explaining customer satisfaction.
Chen & Kao (2010)	OTA	Process quality and outcome quality	Process quality and outcome quality influence customer satisfaction.
Tsang, Lai & Law (2010)	OTA	Customer relationship, safety & security, website functionality, fulfillment & responsiveness, information quality & content, appearance, and presentation	Website functionality, information quality & content, safety & security, and customer relationship influence customer satisfaction.
Hsu, Chang & Chen (2012)	OTA	Perceived flow and perceived playfulness	Perceived flow and customers’ perceived playfulness affect satisfaction.
Ting, Chen & Lee (2013)	OTA	technology acceptance, perceived risk, reduced transaction cost, and service quality	Customers’ e-satisfaction is influenced by service quality and online risk.
Pereira, de Fátima Salgueiro & Rita (2017)	OTA	Online routine, customers’ innovativeness, website’s image perceptions, and online knowledge	Routine, website’s image, and knowledge significantly affect e-customer satisfaction.
Ju et al. (2019)	Airbnb	n/a	The facility produces distinctive, website, and host effects on customer satisfaction.
Sthapit et al. (2020)	Airbnb	Consumption’s values (functional, social, and emotional), co-creation, and information overload	The absence of information overload and co-creation contribute to satisfaction with using the Airbnb website.
Prassida, Hsu & Chang (2021)	OTA and Hotel	Service quality and the perceived value	The perceived value of offline services and online service quality are crucial influence customer satisfaction.

OCRs usually contain text comments and overall ratings. These comments demonstrate customer satisfaction’s attributes, and the overall ratings show customers’ overall satisfaction (Xu, 2020). Tao & Kim (2019) used OCRs to find a new attribute of customer satisfaction which is onshore cruiser experiences attributes. Situmeang, de Boer & Zhang (2020) comprehended customer satisfaction using OCRs and affirmed OCRs can develop a sustainable strategy for the restaurant industry. Based on the above findings, this study utilizes OCRs to discover the vital attributes of OTA customer satisfaction.

### A Text mining approach for pre-processing

Text mining is a knowledge exploration approach that consolidates techniques of natural language processing, information retrieval, machine learning, and data mining (Yang et al., 2018; Zhou & Xue, 2020). The essential task of text mining is to transform texts into numerical data for analysis through natural language processing including editing, analyzing, and organizing an enormous number of texts to provide explicit information (Sullivan, 2001). Previous studies found that text mining was an efficient way to obtain key issues from an enormous number of OCRs and customers’ thoughts can be demonstrated all the more plainly (Xu & Li, 2016; Chiu & Lin, 2018). Compared with manual content analysis, text mining has relevant advantages such as less time and human works to perform analysis (Guo et al., 2016) and extraction of new variables (Hong & Park, 2019).

Text mining techniques have been applied in different subjects particularly in tourism and hospitality research. Jia (2018) proposed a pre-processing process to analyze restaurant customers’ reviews and present insights into the analysis of reviews. Cheng & Jin (2019) identified ‘price’ as a key influencer to Airbnb with a text mining approach on OCRs. This study employs a text mining approach to transform OCRs into numerical data prepared for the feature selection process.

### Feature selection with least absolute shrinkage and selection operator (LASSO)

Feature selection is a process of looking for the best subset of characteristics, from the original set according to the given goal of processing and criteria (Swiniarski & Skowron, 2003). Feature selection has two purposes which are to avoid the curse of dimensionality in modeling and to get important features. Its process is to eliminate unimportant features that can decrease the difficulty of learning tasks (Kwok, Zhou & Xu, 2015). Due to the frequent long length, generous number, and open structure of online textual reviews, extracting key points from textual reviews can be challenging and complex (Gandomi & Haider, 2015). The questions are which features are to be included in the model, and which feature selection algorithms can be employed. The existing solutions of feature selection can be separated into the filter, wrapper, and embedded methods. The filter method is a pre-processing stage and uses criteria not involving any learning machine and, by doing that, it does not consider the impacts of a chosen feature subset (Kohavi & John, 1998; Guyon & Elisseefl, 2006; Lal et al., 2006). The wrapper method assesses a subset of features according to the accuracy of a given predictor (Kohavi & John, 1998; Guyon & Elisseeff, 2003). The embedded methods of feature selection are suitable for the process of training and to give learning machines (Guyon & Elisseeff, 2003). Filter and wrapper methods do not evaluate the feature sets iteratively, in contrast, the embedded method is more robust in over-fitting data (Cai et al., 2018). One typical algorithm of the embedded methods is called the least absolute shrinkage and selection operator (LASSO) (Tibshirani, 1996).

LASSO is a regression method that involves setting the absolute size of the regression coefficients which does regression and feature selection simultaneously to enhance interpretability of the statistical model it produces (Tibshirani, 1996). LASSO forces a limit on the sum of absolute values of the regression coefficients, enabling some coefficients to be zero, exposing unimportant features, so those coefficients of important features are not zero. The principal feature of LASSO is that the pressure factor and the feature selection can be automatically cultivated in the evaluation process (Huang, Wang & Kochenberger, 2017). Through a variable selection procedure with shrinkage of regression coefficients to zero then picking the most fitted coefficients in the linear regression, LASSO controls the model complexity and increases the selection performance (Sant’Anna, Caldeira & Filomena, 2020). Past research confirmed a better result can be accomplished by using LASSO.

Previous research has shown that LASSO outperforms other algorithms in terms of results. Chang et al. (2019) used support vector machines (SVM) and back-propagation neural networks (BPN) to compare LASSO and decision tree (DT) in order to find the most critical un-revisit intention factors, and found that LASSO had higher accuracy than DT. Dastjerdi, Foroghi & Kiani (2019) predicted a manager’s fraud risk and came up with a LASSO result that was much more precise than the Convex Optimization (CVX). After being analyzed by support vector machines (SVM), Chang et al. (2020) discovered that LASSO obtained superior accuracy compared to support vector machines recursive feature elimination (SVM-RFE) in order to determine the most important factors toward customers’ trust in O2O models. This study employs LASSO to do feature selection due to its powerful algorithm which enables to get the most important variables of the OTA customer satisfaction from OCRs.

## Research Methods

**Figure 1:**
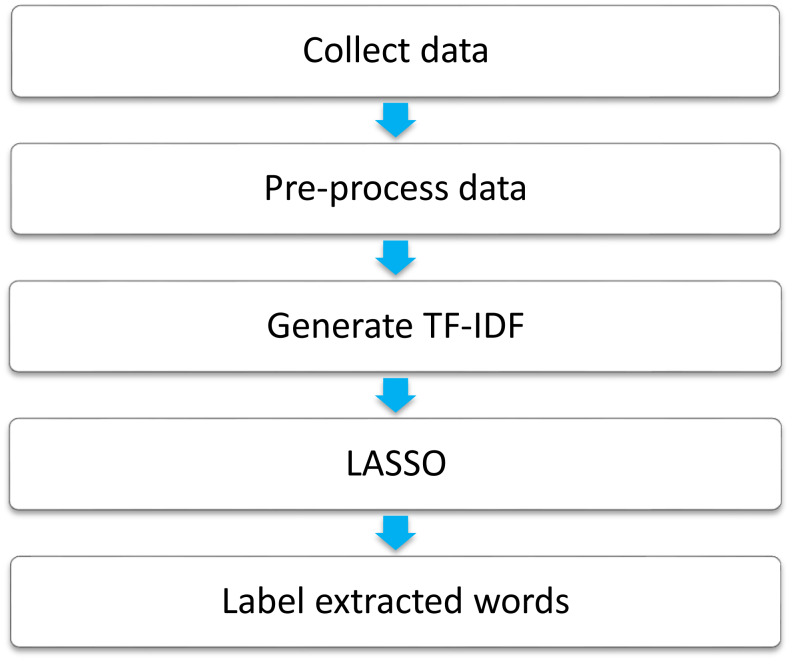
Steps of feature selection.

In line with previous studies (Chang et al., 2020; Chen et al., 2021), the feature selection consists of the following five steps; (1) data collection, (2) data pre-processing, (3) generate TF-IDF, (4) Lasso, and (5) words labeling (Fig. 1). Details of the process are described as follows.

### Data collection

This study collected and analyzed customers’ opinions toward a well-known OTA because it operates worldwide (Trefis Team, Great Speculations, 2019). This OTA was available in 43 languages and offered 28 million accommodations at 15 thousand destinations in 226 countries and territories in December 2020. OCRs were considered as a source of data because they reflected alternative customers’ perceptions. Trustpilot.com (https://www.trustpilot.com/) is an open online platform for evaluating services, companies, or brands by customers. Trustpilot.com provided more OCRs than other similar websites during the data collection period. Singh & Söderlund (2020) also collected retailers’ OCRs in the UK from Trustpilot.com. Therefore, the OTA customers’ reviews on Trustpilot.com posted in English were chosen as samples in this study. To ensure these reviews represent the majority of customers’ voices, Chiu & Lin (2018) suggested that the minimum reviews have better results with more than 50 samples. A total of 1,313 OCRs with comment texts and overall ratings (1–5 scores) were obtained, from March to August in 2020 during the COVID-19 outbreak.

### Data pre-processing

Online customer reviews commonly appear with long sentences. In order to get fewer words but probably more important words. The TF-IDF process was applied to clean the sentences into pieces by pieces of words based on their occurrences. Along the process, the words with low occurrence would be removed. The data pre-processing was performed by the data analytics software, namely RapidMiner Studio^®^ 9.4r. A tokenization function was applied to remove unrelated characters, symbols, emoticons, and stop words, such as “the”, “are”, “that”, etc., and to reorganize the texts into lowercase letters. This function was also used to avoid words less than three letters that could not provide enough significant information, such as “on”, “at”, “no”, etc. The texts were tokenized with non-letter separators that separated the comments into small pieces. Further, a stem method was applied to the root of the token, for example, “simplistic” and “simplicity” were purified into the single token “simple” resulting in a single meaning of words. Segment corpus with bigram in which two words were often found together throughout the document, such as “full_refund”, “excellent_service”. Then a pruning method was applied by which any words appearing less than five times in the dataset were removed because these words were mentioned less which meant having a less significant contribution to the model. Finally, the term frequency-inverse document frequency (TF-IDF), the relative frequency of a certain word in a specific document (Ramos, 2003; Sezgen, Mason & Mayer, 2019), was ready to be analyzed. TF-IDF was confirmed to be an effective method for word weighting in information retrieval (Sebastiani, 2002). Sezgen, Mason & Mayer (2019) applied TF-IDF to deal with online customer reviews to analyze the reviews further.TF-IDF is defined as follows.

(1) \begin{eqnarray*}id{f}_{i}=\log \nolimits 2( \frac{N}{{n}_{i}} )+1.\end{eqnarray*}

TF-IDF (weighted) score is calculated by;

(2) \begin{eqnarray*}{w}_{ij}=t{f}_{ij}\times id{f}_{i}.\end{eqnarray*}

In Eq. (1), N = the number of total documents and n_i_ = the term frequency of term i in the overall documents. In Eq. (2), tf_ij_ refers to the number of occurrences of term i in document j and idf_*i*_ represents the general significance of term i in the overall documents. TF-IDF is a metric that multiplies the two quantities tf and idf. This method was applied to weight which words were most frequently shown in one single review. When a word’s TF-IDF score is higher, it demonstrates the word appears frequently in the part of documents (Chen et al., 2016; Sebastiani, 2002). The most frequent words would be analyzed further. In this study, TF-IDF was used to calculate the weights of words in a document. Finally, a TF-IDF weighted with 5,409 (selected words) × 1,313 (data samples) term-by-document matrix was established. The TF-IDF result was used by LASSO for selecting the important words.

Trustpilot.com allows customers to give overall ratings from 1 to 5 scores for a subject. Farhadloo, Patterson & Rolland (2016) transformed the ratings into a binary scale, with an overall rating score of 1, 2, or 3 being marked 0 and scores of 4 or 5 being marked 1. This method converts the 5-point scale into a 2-point binary scale representing bad *versus* good satisfaction (0 = unsatisfied and 1 = satisfied), and its robustness was confirmed by previous studies (Atalık, Bakır & Akan, 2019; Tao & Kim, 2019). The dependent variable in the LASSO method adopted the binary mode (zero and one) which is more precise and powerful than the continuous-dependent mode (Dastjerdi, Foroghi & Kiani, 2019). In this study, a binary method was used to mirror customer satisfaction’s scores.

### LASSO

Once the TF-IDF was established, LASSO was run by Matlab^®^ software. It performed regression and feature selection functions simultaneously to extract the significant features considering the following selection criteria, as shown in Eq. (3), where x is the explanatory variable, T is the number of data and *λ* is the adjustment coefficient.

(3) \begin{eqnarray*}\min \nolimits =\sum _{t=1}^{T}({\mathrm{y}}_{\mathrm{ t}}-{\beta }_{0}-{\beta }_{1}{\mathrm{x}}_{1,\mathrm{t}}-\ldots -{\beta }_{\mathrm{k}}{\mathrm{x}}_{\mathrm{k},\mathrm{t}})^{2},~\mathrm{s}.\mathrm{t}.~\sum _{\mathrm{j}=1}^{\mathrm{k}} \left\vert {\beta }_{\mathrm{ j}} \right\vert \leq \lambda .\end{eqnarray*}

According to Eq. (3), a regression parameter value namely *β*i is limited by a specific penalty selection benchmark, and afterward, the suitable variables are chosen. Given a k-explained transformation, the parameter estimate ‘\hat{\beta}’ is influenced by the value of *λ*. When the *λ*’s value approaches infinity, the estimate of the parameter \hat{\beta} is not limited, and the estimate is the value determined by the least-squares method. The contrary situation is when the *λ* is adjusted to 0, all parameter estimates become 0. The explanatory variable *x*, which is closely connected with *y*_*t*_, would vary and differ from zero as the value of *λ* is gradually increased from zero, suggesting that the explanatory variable is significant. As a result, in this experiment, the premise for finding essential features is to see if the coefficient is 0, and if the coefficient is not 0, the feature is considered significant.

### Identifying factors of customer satisfaction

After gathering the relevant keywords with LASSO, the following step was to classify them using a five-fold cross-validation experiment to establish the important factors based on their frequency of occurrence. The essential idea of the five-fold cross-validation experiment is that the sample data set is randomly partitioned into five mutually exclusive subsets (the folds). The technique was carried out in stages, with one subset serving as a testing subset and the other four serving as training subsets, and it ran in turn. While the group experiment approach was not relevant during the procedure, the five-fold cross-validation experiment ensured that every measurement was used for the objectives of training, testing, and validating. The five-fold cross-validation experiment was used to rank the important words based on their occurrence frequencies. When a word appears more times the more significant the word is Lim & Kim (2020). Chang et al. (2019) and Chang et al. (2020) applied a five-fold cross-validation experiment to rank selected features.

## Experimental Results

### LASSO results

In the parameter setting of LASSO, built-in functions in Matlab^®^ were employed to filter out the essential words. would impose some words’ regression coefficients to zero which means these words are not relevant to the regression model (Zhao & Yu, 2006; Makarov et al., 2019; Wang, 2021). Simply put, the words with regression coefficients zero were considered as not important words to influence customer satisfaction. Whereas, words with regression coefficients that are not zero can be considered as important words to influence customer satisfaction (Zhang & Huang, 2008). Since the five-fold cross-validation experiment approach was applied, the dataset was split into five equivalent parts. The five parts were run each by parameter setting of LASSO. With a five-fold cross-validation experiment approach, the results were also obtained five results as shown in Table 2 which is Fold#1, Fold#2, so on.

**Table 2:** LASSO Results.

Extracted keywords	Fold#1	Fold#2	Fold#3	Fold#4	Fold#5	Frequency
thank_you	2.5884113	2.5038914	2.4260114	2.5038914	2.5038914	5
never_had	2.3115838	2.0744914	1.9410475	2.0744914	2.0744914	5
great	1.8180118	1.7606125	1.7277082	1.7606125	1.7606125	5
excellent	1.2155508	1.1060514	0.9965587	1.1060514	1.1060514	5
my_first	1.1209985	0.5486933	0.2081031	0.5486933	0.5486933	5
easy	1.0169599	0.9260635	0.8703357	0.9260635	0.9260635	5
perfect	0.9039296	0.6524267	0.510088	0.6524267	0.6524267	5
impress	0.8595221	0.6132996	0.4602385	0.6132996	0.6132996	5
back_for	0.8271636	0.5089204	0.3158973	0.5089204	0.5089204	5
quick	0.8150146	0.6692242	0.5879905	0.6692242	0.6692242	5
fantastic	0.7642072	0.5911542	0.500492	0.5911542	0.5911542	5
within_minute	0.7637819	0.3467163	0.0515449	0.3467163	0.3467163	5
amazing	0.7536879	0.5683715	0.4642053	0.5683715	0.5683715	5
continue	0.7123623	0.4945143	0.3565506	0.4945143	0.4945143	5
and_help	0.6940792	0.3949878	0.2057348	0.3949878	0.3949878	5
thank	0.5552965	0.506089	0.481362	0.506089	0.506089	5
full_refund	0.5384723	0.3863128	0.2904346	0.3863128	0.3863128	5
great_service	0.4513193	0.2482341	0.1315517	0.2482341	0.2482341	5
refundable_hotel	0.448031	0.2503497	0.1319644	0.2503497	0.2503497	5
bit	0.435254	0.1913623	0.0425053	0.1913623	0.1913623	5
had	0.4052053	0.2911124	0.2136364	0.2911124	0.2911124	5
was_able	0.341869	0.2191523	0.1510289	0.2191523	0.2191523	5
none	0.3339257	0.1824334	0.0834002	0.1824334	0.1824334	5
were_verified	0.7820855	0.2170775	0	0.2170775	0.2170775	4
bad_review	0.4358002	0.0438122	0	0.0438122	0.0438122	4
cheap	0.3323283	0.1161286	0	0.1161286	0.1161286	4
had_book	0.1646458	0.0196089	0	0.0196089	0.0196089	4
good	0.1159964	0.0376419	0	0.0376419	0.0376419	4
so_far	0.3159153	0	0	0	0	1
did_so	0.2995969	0	0	0	0	1
and	0.2195096	0	0	0	0	1
comfort	0.1994573	0	0	0	0	1
last_minute	0.162742	0	0	0	0	1
excellent_service	0.155303	0	0	0	0	1
custom	0.1426258	0	0	0	0	1
my_behalf	0.0746737	0	0	0	0	1
overwhelming	0.0629221	0	0	0	0	1
only	0.0615804	0	0	0	0	1
best	0.0542489	0	0	0	0	1
other_companies	0.0490777	0	0	0	0	1
little	0.008604	0	0	0	0	1

### Identifying factors of customer satisfaction

After the significant words of customer satisfaction were identified by LASSO, the essential words were ranked by their occurrences using the five-fold cross-validation experiment. The occurrence refers to how many times the words appear in the five experiments. As listed in Table 2, this study only obtained 5, 4, and 1 times of word occurrence frequency following LASSO regulations. If the words with coefficient were not zero showed up more within 5 experiments, it inferred the words were more significant. To diminish subjectivity in word labeling, those words that had similar meanings, purposes, and frequencies were gathered together. This method is simple and objective. Results showed that refunds, promptness, easiness, and assurance were the first-ranked factors placed in the code F1. Bad reviews and cheap were the second-ranked factors placed in the code F2. Excellent service and comparison were the third-ranked factors placed in the code F3. However, experiences were not categorized into a factor because customers showed their experiences with non-meaningful words. Due to the sentiment words only showing gladness and disappointment without meaningful information, it was also not categorized as a factor. Table 3 lists the factors after the words are labeled and ranked based on their occurrences.

## Discussion

Refunds, promptness, easiness, and assurance were found as first-ranked factors to OTA customer satisfaction in this study. The refund became a thorny problem to OTAs during the COVID-19 pandemic (Connor, 2020; Piccinelli, Moro & Rita, 2021). Many airline and hotel customers had to cancel tickets and bookings but some went through complicated refund processes (Uğur & Akbıyık, 2020; Piccinelli, Moro & Rita, 2021). Customers need an easy and agile process for the booking and refunding process (Tsang, Lai & Law, 2010). Promptness is important during the COVID-19 pandemic because travelers can become dissatisfied if the requests are not served within the allowed time (Lee & Ko, 2021). Easy process is required by travelers when they requested services, especially during the COVID-19 pandemic (Foroudi, Tabaghdehi & Marvi, 2021). Assurance was also found as an important factor for travelers, and it was always during the pandemic as Uğur & Akbıyık (2020) stated during the pandemic, travelers want tourism providers to give them assurance services.

**Table 3:** Associated factors with customer satisfaction.

Frequency	Code	Factors	Words
5	F1	Refunds	full_refund, refundable_hotel
		Promptness	quick, within_minute
		Easiness	easy
		Assurance	and_help
		Experiences	my_first, never_had, was_able
		Sentiment	thank_you, great, excellent, perfect, impress, fantastic, amazing, great_service, thank, bit, had, none, back_for, continue
4	F2	Bad reviews	bad_review
		Cheap	cheap
		Experiences	were_verified, had_book
		Sentiment	good
1	F3	Excellent service	excellent_service
		Comparison	other_companies
		Experiences	last_minute, custom, only, little, so_far, did_so, and
		Sentiment	comfort, overwhelming, my_behalf, best

Bad reviews and cheap were found as the second-ranked factors in this study. Previous studies suggested that customers’ comments either negative or positive are influenced by customer satisfaction (Berezina et al., 2016; Xu, 2020). This study found negative reviews as the second-ranked factor to customer satisfaction. It is an alert to OTAs that customers’ negative comments have greater impacts on potential travelers than those positive messages (Rianthong, Dumrongsiri & Kohda, 2016; Sánchez-Franco, Navarro-García & Rondán-Cataluña, 2019). Negative comments for hospitality and tourism industries possibly impair OTAs’ reputations and block orderings from the existing and future customers during the COVID-19 outbreak (Luo & Xu, 2021). Cheap was an important factor for customer satisfaction because most travelers were used to searching for bargain products or services among OTAs during the COVID-19 outbreak (Nilashi et al., 2022).

Excellent service and comparison were the third-ranked factors. Quality service is always the first priority for customers. During the pandemic, travelers are used to comparing offerings among OTAs and choosing the best one (Nilashi et al., 2022). During the pandemic, choosing excellent services with comparing offerings among OTAs became a priority for travelers (Nilashi et al., 2022).

Overall, this study contends that external factors other than core services, such as negative reviews and comparison, have an impact on customer satisfaction. These findings differ from those of previous studies (Table 1) which found that only internal factors have a positive influence on customer satisfaction. On the other hand, this study confirms that internal factors have a significant impact on customer satisfaction.

The coronavirus pandemic has influenced industries worldwide and tested companies’ capabilities to manage the crisis. It has changed individuals’ traveling behavior, OTAs’ marketing programs must align with this trend. This study reveals a new set of critical factors to OTA customer satisfaction during the COVID-19 pandemic which informs traveling industries to transform their customer satisfaction’s indicators.

## Conclusion

This study empirically examines the critical factors of customer satisfaction toward online travel agencies when COVID-19 happened in the world. Based on the online customer reviews during the COVID-19 pandemic, a text mining method including the LASSO approach was used to extract the significant factors of customer satisfaction toward OTAs. This approach is feasible to explore extensive issues for travel industries.

During the COVID-19 outbreak, many OTAs have endured great losses from the shortage of orders and faced a bleeding bottom-line of the financial situation. This study helps OTAs to re-examine their service priorities in order to do trade-off offerings. Regarding the questions of what are the most and critical attributes of customer satisfaction and also the ranking of those attributes. Refunds, promptness, easiness, and assurance were on the first-ranked, followed by bad reviews & cheap in the second-ranked and excellent service & comparison in the third-ranked list. Refunds, bad reviews, assurance, and comparison are ranked as novel factors of customer satisfaction. Understanding the new set of customer satisfaction factors provides insights for OTAs. Managers may place the first-ranked factors to be the top list of their services. Therefore, the generalization of results to other OTAs should be cautious.

Facing the global recession in the tourism industry caused by COVID-19, it is suggested that OTAs redesign competitive offerings to stimulate customer satisfaction during and post-pandemic crises. Second, OTAs should coordinate with tourism suppliers to make easy and fast refund policies with assurance service and procedures for customers. Also, OTAs can re-examine their competitive positions through OCRs, especially good and bad reviews.

Online customer reviews are a valuable source for hospitality and tourism research, their applications are still under-investigated. A limitation of this study is solely collecting OCRs to an OTA from a single review website. To improve the external validity of results, future studies can collect OCRs of multiple online traveling agencies.

##  Supplemental Information

10.7717/peerj-cs.850/supp-1 Supplemental Information 1 Raw data

10.7717/peerj-cs.850/supp-2 Supplemental Information 2 MATLAB code

## Additional Information and Declarations

### Data Availability

The following information was supplied regarding data availability: The data was retrieved from  https://www.trustpilot.com/review/www.booking.com and is available as a Supplemental File. The MATLAB code is available as a Supplemental File.
